# *Mycobacterium heraklionense* hand tenosynovitis—a case description of a three-year treatment course and perioperative measurement of azithromycin target tissue concentrations

**DOI:** 10.1128/aac.01670-24

**Published:** 2025-03-17

**Authors:** Mads Kristian Duborg Mikkelsen, Andrea René Jørgensen, Victor Naestholt Dahl, Christian Morberg Wejse, Mats Bue, Maiken Stilling

**Affiliations:** 1 Aarhus Denmark Microdialysis Research (ADMIRE), Orthopaedic Research Laboratory, Aarhus University Hospital 11297 , Aarhus, Denmark; 2 Department of Clinical Medicine, Aarhus University 11291 , Aarhus, Denmark; 3 Department of Infectious Diseases, Aarhus University Hospital 11297 , Aarhus, Denmark; 4 Department of Orthopaedic Surgery, Aarhus University Hospital 11297 , Aarhus, Denmark; Houston Methodist Hospital and Weill Cornell Medical College, Houston, Texas, USA

**Keywords:** nontuberculous mycobacteria, microdialysis

## Abstract

In a *Mycobacterium heraklionense* tenosynovitis case, we observed that azithromycin-loaded calcium sulfate beads placed in the surgical wound had minimal systemic absorption and achieved over 100-fold higher target tissue concentrations than a systemic azithromycin-based treatment regimen alone. However, continued excessive wound drainage necessitated surgical removal of the beads. While systemic antibiotics and surgery remain central to nontuberculous mycobacterial treatment, the role of local antibiotic administration in reducing the bacterial burden warrants further investigation.

## INTRODUCTION

Extrapulmonary nontuberculous mycobacterial (NTM) infections are notoriously difficult to treat, and in recent years, NTM hand infections have received increasing attention (1). *Mycobacterium heraklionense,* a member of the *M. terrae* complex, was first described by Tortoli et al. in 2013 (2). Since then, multiple hand infection cases have been documented, with a predominance of tenosynovitis (3). Azithromycin is a cornerstone in treating several NTM species; however, little is known about the relationship between azithromycin plasma and tissue concentrations and the therapeutic effect. In the following section, we present the case of an immunocompetent female in her mid-60s undergoing treatment for *M. heraklionense* hand tenosynovitis, focusing on perioperative azithromycin target tissue concentrations.

## CASE PRESENTATION

**Fig 1:**
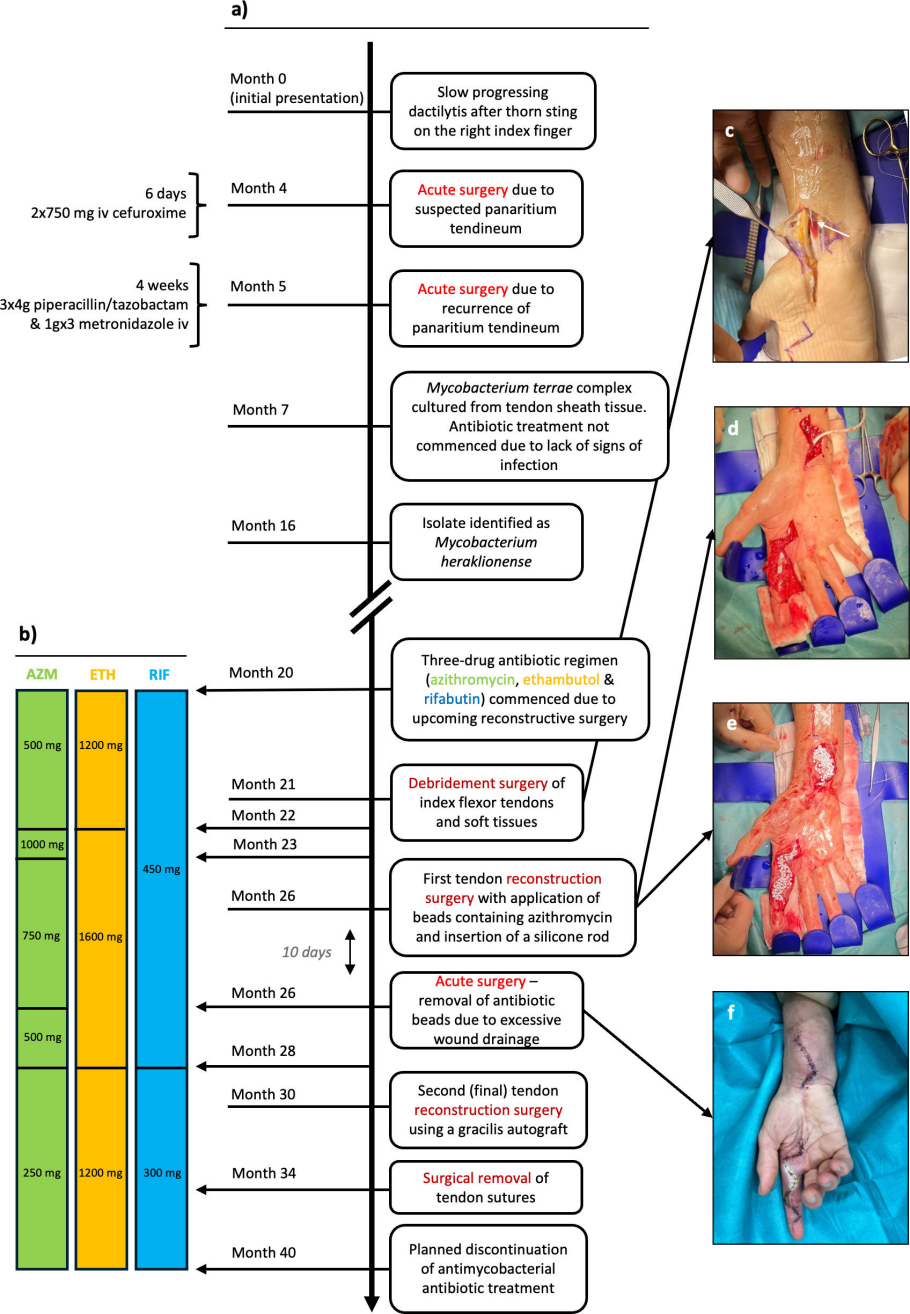
Case timeline with clinical images during the treatment. (**a**) Timeline of disease progression and surgical interventions. (**b**) Timeline of antimycobacterial treatment, with administered daily doses. (**c**) Placement of a microdialysis catheter (white arrow) in the subcutaneous tissue of the distal forearm during debridement surgery. (**d**) Placement of a silicone rod during the first reconstructive surgery. (**e**) Application of azithromycin-containing beads in the distal forearm and index finger during the first reconstructive surgery. (**f**) Inflamed wound with persistent drainage 10 days after implantation of the azithromycin-containing beads. AZM, azithromycin; ETH, ethambutol; RIF, rifabutin.

A female in her mid-60s without any relevant medical history, aside from small joint hand osteoarthritis, presented to her local clinic with slowly progressing swelling and soreness of the right index finger (Fig. 1a). Suspectedly, this was caused by a thorn prick from a barberry bush. Four months later, her symptoms had worsened, with intense pain, paresthesia, and marked swelling of the finger, though no systemic signs of infection were present. Acute surgery was performed for suspected infectious tenosynovitis, which involved debridement of infected and necrotic tissue alongside median nerve decompression. Perioperative biopsy samples from the tendon sheath, pulley, and subcutaneous tissue were negative on routine bacterial cultures. One month later, symptoms returned, prompting another acute surgery, which revealed deposits of pus between the tendon floor and the bone, along with pulley destruction and massive tendon sheath granulation (5 mm thick). No rice body formation was observed. Extensive debridement of infected and necrotic tissue was performed, and histopathological examination of tissue biopsies from the tendon sheath and pulleys demonstrated necrotizing granulomatous inflammation but no acid-fast bacilli (Ziehl–Neelsen staining). After acute surgery, symptoms of infection quickly resolved. Paresthesia of the index finger persisted, and bowstringing of the flexor tendons with adhesion developed. Seven months after clinical presentation, mycobacterial cultures from the tissue biopsies in the first surgery grew a few colonies of *M. terrae* complex. Auramine–rhodamine fluorochrome staining was negative. Antimycobacterial treatment was not commenced due to the absence of clinical signs of infection and seemingly spontaneous improvement.

Sixteen months after the initial presentation, the International Reference Laboratory of Mycobacteriology in Copenhagen identified *M. heraklionense* as the causative species from the earlier cultured isolate using whole-genome sequencing. Two months later, drug susceptibility testing results were available (Table 1). A two-stage tendon reconstructive surgery was planned to address bowstringing of the flexor tendons and tendon adhesion.

**TABLE 1:** Susceptibility profile of the *Mycobacterium heraklionense* isolate based on the proportion method using Mycobacterium Growth Indicator Tube (MGIT) and Löwenstein–Jensen medium, with MIC interpretations according to Clinical and Laboratory Standards Institute guidelines

Drug	Minimal inhibitory concentrations, µg/mL	Interpretation
Amikacin	16	R
Clarithromycin	8	S
Ethambutol	5	S
Levofloxacin	2	R
Moxifloxacin	1	R
Linezolid	16	R
Rifabutin	0.5	S
Rifampicin	1	R

## CHALLENGE QUESTION

What is the optimal antibiotic treatment duration for hand tenosynovitis caused by an NTM infection when a foreign body has been inserted?

(A) The presence of a foreign body should not influence treatment duration, and a 6-month treatment is sufficient.

(B) Caution is warranted due to the presence of a foreign body. A treatment duration of more than 1 year after insertion is preferable, while 3-6 months may be sufficient if the foreign body is removed.

(C) The treatment regimen should be guided by the clinical course and discontinued only when there is a clinical conviction of cure.

(D) Option C, but additional local therapies (e.g., surgery, negative pressure wound therapy with instillation and dwell time with topical antibiotics (NPWTi-d), antibiotic-loaded beads) should be considered to improve infection control and potentially shorten the treatment duration.

## TREATMENT AND OUTCOME

The treatment regimen and duration were discussed at a multinational, multidisciplinary meeting (4). Based on this, a combination regimen of azithromycin, rifabutin, and ethambutol, initiated one month before tendon reconstruction surgery, was recommended to continue for 6 months after the final surgical intervention (Fig. 1b). Antibiotic dosing was guided by therapeutic drug monitoring in plasma, leading to dose adjustments throughout treatment.

After 21 months, surgery revealed massive tendon adhesion and yellow low-viscosity pus-like deposits in the tendon–bone interface. Thorough debridement was performed, including excision of the flexor tendon and median nerve decompression. Intraoperatively, microdialysis catheters were placed (Fig. 1c) in the flexor carpi radialis tendon sheath, the brachioradial muscle, and forearm subcutaneous tissue to enable postoperative sampling (3 hours) of steady-state azithromycin concentrations in target tissues. Biopsies from the tendon, pus-like deposits, and bone were sent for routine bacterial and mycobacterial culturing but showed no bacterial growth.

The first-stage tendon reconstruction was performed 26 months after the initial presentation, with implantation of a silicone rod (Hunter I) (Fig. 1d). Given the risk of biofilm formation due to the presence of a foreign body in a formerly infected wound, 3 cc of antibiotic calcium sulfate beads (STIMULAN Rapid Cure; Biocomposites, UK) prepared with 300 mg of azithromycin was placed around the silicone rod (Fig. 1e). Microdialysis catheters were positioned along the silicone rod in the index finger and above the wrist, as well as in the flexor carpi radialis tendon sheath, the brachioradialis muscle, the forearm subcutaneous tissue, and the radius cancellous bone for postoperative sampling (10 hours) of steady-state azithromycin concentrations. However, continuous wound drainage necessitated surgical removal of the beads 10 days after implantation (Fig. 1f).

The second-stage flexor digitorum profundus tendon reconstruction was performed after 30 months using a gracilis tendon autograft. Eleven months after the final reconstructive surgery, the patient showed no signs of infection.

### Methods

Microdialysis was used to sample local unbound (free) steady-state azithromycin concentrations in target tissues (5). Plasma samples were collected at the midpoint of each sampling interval. Microdialysis catheters type 70 with 20 mm and 10 mm membrane lengths, and a 20 kDa molecule cutoff (M Dialysis AB, Stockholm, Sweden) were used. The catheters were perfused with 0.9% NaCl at a 1 µL/min flow rate using a CMA 107 precision pump. At the end of each sampling period, retrodialysis with azithromycin solutions of 10 µg/mL and 1,000 µg/mL was performed to calibrate the system (6). Azithromycin concentrations were quantified using high-performance liquid chromatography.

### Results

**Fig 2:**
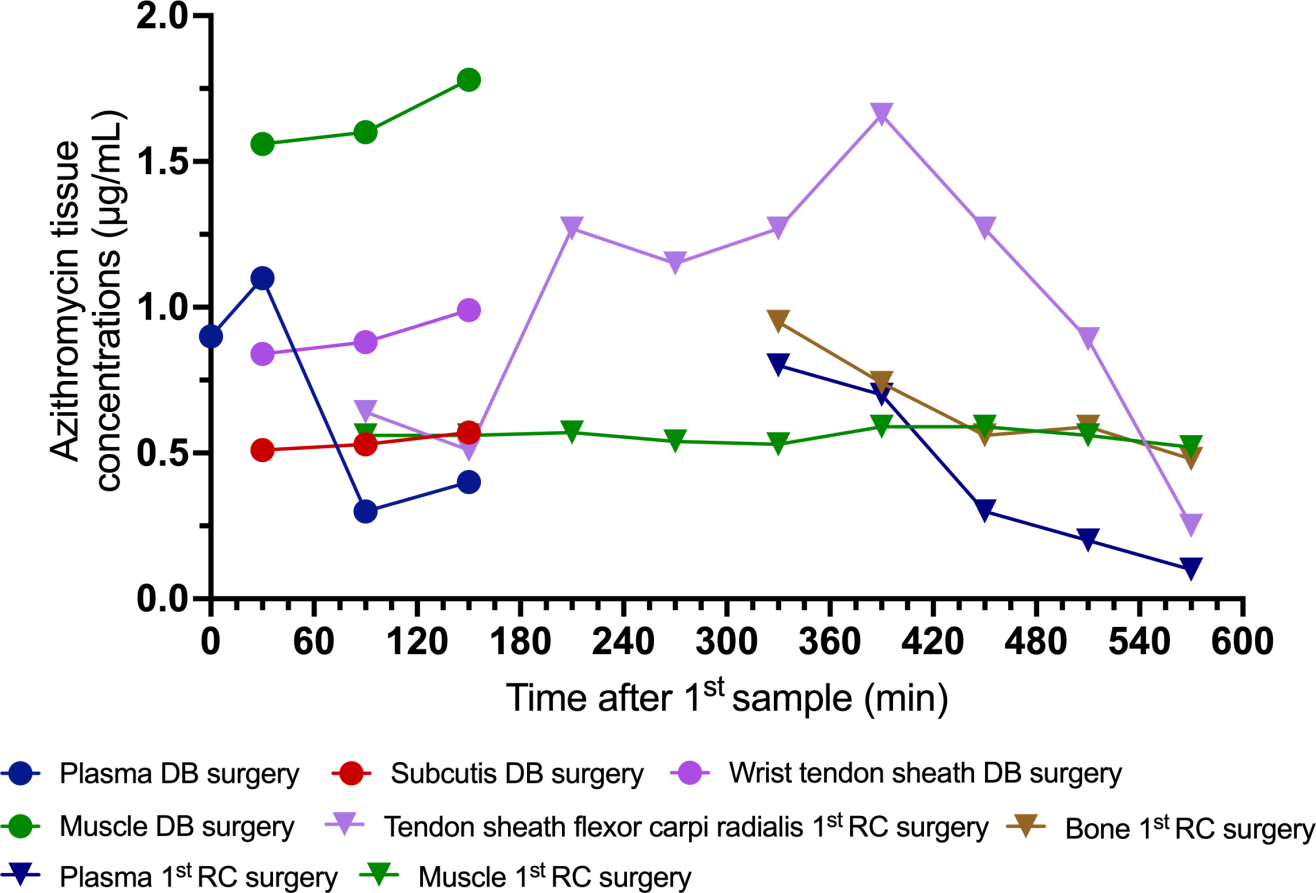
Azithromycin concentrations in plasma and target tissues, measured in relation to the debridement (DB) surgery and the first tendon reconstructive (RC) surgery.

Following the debridement surgery, the free concentrations of azithromycin measured with microdialysis in muscle, subcutaneous tissue, and the tendon sheath ranged from 0.5 to 1.8 µg/mL (Fig. 2). Plasma azithromycin total concentrations ranged from 0.3 to 1.1 µg/mL (Fig. 2). During the first reconstructive surgery, free azithromycin tissue concentrations in compartments not directly exposed to the azithromycin-loaded beads ranged from 0.3 to 1.7 µg/mL (Fig. 2). In contrast, tissues in direct contact with the beads had free azithromycin concentrations almost 100-fold higher, ranging from 56 to 151 µg/mL. Plasma azithromycin total concentrations remained between 0.1 to 0.8 µg/mL (Fig. 2), resembling those observed following debridement surgery, indicating that systemic azithromycin concentrations were unaffected by the local azithromycin treatment.

### Discussion

This case describes a three-year treatment course with documentation of perioperative azithromycin tissue concentrations in a patient with *M. heraklionense* hand tenosynovitis. Treatment involved surgical insertion of azithromycin-containing beads in addition to conventional systemic antibiotic therapy. A regimen consisting of azithromycin, rifabutin, and ethambutol was initiated one month before tendon reconstruction surgery and continued for 20 consecutive months.

Microdialysis revealed comparable perioperative azithromycin concentrations in plasma and various target tissues (muscle, tendon sheath, bone, and subcutaneous tissue) that were not in direct contact with the azithromycin-containing beads. These concentrations exceeded previously suggested C_max_ plasma targets of ≥0.4 and >0.2 µg/mL used in clinical studies of patients with *M. avium* complex lung disease (7, 8). Although these targets may not be directly applicable due to variations in species and disease manifestation, they serve as a valuable starting point in the absence of more robust alternatives. Placing azithromycin-containing beads during surgery resulted in high azithromycin concentrations in tissues directly adjacent to the beads, with tissue concentrations ranging from 56 µg/mL to 151 µg/mL. Unfortunately, the beads had to be surgically removed after 10 days due to excessive wound drainage from the index finger, which prevented wound healing and posed a risk of superinfection. Most likely, the thin skin flaps on the finger did not provide sufficient tissue coverage to seal the fluid production caused by the dissolution of the calcium sulfate beads. Despite the challenges associated with assessing the clinical impact of short-term azithromycin exposure, it may have reduced the perioperative mycobacterial load in comparison to systemic treatment alone. Nevertheless, such an assertion remains speculative, and it is not possible to ascertain the clinical efficacy of the local therapy based solely on this case.

For NTM hand infections, a combination of antibiotics is typically administered over many months, with or without surgery (1). Nevertheless, there is a lack of comprehensive clinical evidence to inform treatment protocols and the optimal duration of therapy (9). Recently, Stemkens et al. successfully incorporated topical antibiotic treatment (NPWTi-d) alongside systemic antibiotics in three similar cases of skin and soft tissue mycobacterial infections (10). In our case, we tried to enhance the understanding of specific tissue concentrations in treating NTM tenosynovitis while also implementing a novel mode of antibiotic delivery. Together, these cases offer intriguing, albeit experimental, perspectives on how topical antibiotics could reduce the local mycobacterial burden to supplement systemic treatment in NTM infections. However, applying localized therapeutic strategies necessitates additional refinement to establish their safety and efficacy thoroughly.

The current pharmacokinetic studies of azithromycin in clinical settings involving NTM infections have focused on patients with lung disease caused by the *M. avium* complex, a rapidly growing mycobacterium (7, 8). In contrast, *M. heraklionense* is a slow-growing mycobacterium, which complicates the correlation of treatment targets, as the minimal inhibitory concentration (MIC) values for macrolides (i.e., clarithromycin) are typically higher for rapidly growing mycobacteria (11). In this case, the isolate’s MIC for clarithromycin, a class drug used for drug susceptibility testing of NTM, was determined to be 8 µg/mL and considered susceptible (11, 12). However, the use of clarithromycin susceptibility testing as a surrogate for azithromycin has been questioned (12). *In vivo* concentrations higher than this MIC were only achieved when azithromycin was administered locally. The calcium sulfate beads may have influenced the intracellular uptake in neutrophils and macrophages, which serve as the primary reservoir, noting that pH and calcium levels significantly regulate this uptake (13). It remains uncertain whether this influence has compromised the efficacy of the antibiotic.

No quantification of coadministered rifabutin or ethambutol concentrations was obtained in this case, making it difficult to evaluate synergistic or antagonistic treatment effects. However, in the treatment of *M. avium* complex, which may be an imperfect comparison, rifamycins and ethambutol are primarily used as companion drugs to prevent the development of macrolide resistance (14).

### Conclusion

This case highlights the challenges of diagnosing and treating NTM hand tenosynovitis. Systemically administered azithromycin may achieve adequate target site concentrations with comparable steady concentrations in target hand tissue and plasma. Still, this is highly reliant on the intricacies of the target discussion. A 100-fold higher azithromycin target tissue concentration can be achieved with local treatment. Nonetheless, using antibiotic calcium sulfate beads as a carrier should be used with caution in anatomical sites with thin skin flaps, such as the fingers, since excessive wound drainage may complicate wound healing and pose a risk of superinfection. While systemic antibiotics and surgery remain central to NTM treatment, the role of local antibiotic administration in reducing bacterial burden warrants further investigation.

## References

[1] Tabaja H, Saleem HY, Bakri K, Tande AJ. 2024. Two decades of insights into nontuberculous mycobacterial hand infections. Open Forum Infect Dis 11:ofae152. doi:10.1093/ofid/ofae152 38651140 PMC11034953

[2] Tortoli E, Gitti Z, Klenk H-P, Lauria S, Mannino R, Mantegani P, Mariottini A, Neonakis I. 2013. Survey of 150 strains belonging to the Mycobacterium terrae complex and description of Mycobacterium engbaekii sp. nov., Mycobacterium heraklionense sp. nov. and Mycobacterium longobardum sp. nov. Int J Syst Evol Microbiol 63:401–411. doi:10.1099/ijs.0.038737-0 22447702

[3] Dutronc H, Sawaya E, Poursac N, Desclaux A, Ménard A, Peuchant O. 2023. Mycobacteriumheraklionense as an emerging cause of tenosynovitis. J Microbiol Immunol Infect 56:197–199. doi:10.1016/j.jmii.2022.08.019 36137925

[4] Dahl VN, Burke A, Fløe A, Bruchfeld J, Schön T, Wejse CM, Andersen AB, Svensson E, van Ingen J, Davies Forsman L. 2024. Advantages and limitations of virtual multi-disciplinary team meetings on difficult-to-treat mycobacteria. Int J Tuberc Lung Dis 28:212–213. doi:10.5588/ijtld.23.0551 38563342

[5] Joukhadar C, Müller M. 2005. Microdialysis: current applications in clinical pharmacokinetic studies and its potential role in the future. Clin Pharmacokinet 44:895–913. doi:10.2165/00003088-200544090-00002 16122279

[6] Kho CM, Enche Ab Rahim SK, Ahmad ZA, Abdullah NS. 2017. A review on microdialysis calibration methods: the theory and current related efforts. Mol Neurobiol 54:3506–3527. doi:10.1007/s12035-016-9929-8 27189617

[7] van Ingen J, Egelund EF, Levin A, Totten SE, Boeree MJ, Mouton JW, Aarnoutse RE, Heifets LB, Peloquin CA, Daley CL. 2012. The pharmacokinetics and pharmacodynamics of pulmonary Mycobacterium avium complex disease treatment. Am J Respir Crit Care Med 186:559–565. doi:10.1164/rccm.201204-0682OC 22744719

[8] Jeong B-H, Jeon K, Park HY, Moon SM, Kim S-Y, Lee S-Y, Shin SJ, Daley CL, Koh W-J. 2016. Peak plasma concentration of azithromycin and treatment responses in Mycobacterium avium complex lung disease. Antimicrob Agents Chemother 60:6076–6083. doi:10.1128/AAC.00770-16 27480854 PMC5038230

[9] Nguyen DC, Buettner AM, Dousa KM. 2023. Case commentary: snother prong of attack? Topical antibiotic instillation with negative pressure wound therapy for nontuberculous mycobacterial skin and soft tissue infections. Antimicrob Agents Chemother 67:e0104823. doi:10.1128/aac.01048-23 38014943 PMC10720531

[10] Stemkens R, Cobussen M, de Laat E, Hoefsloot W, van Crevel R, Aarnoutse RE, van Ingen J. 2023. Successful addition of topical antibiotic treatment after surgery in treatment-refractory nontuberculous mycobacterial skin and soft tissue infections. Antimicrob Agents Chemother 67:e0078823. doi:10.1128/aac.00788-23 38014946 PMC10720519

[11] Khare R, Brown-Elliott BA. 2023. Culture, identification, and antimicrobial susceptibility testing of pulmonary nontuberculous mycobacteria. Clin Chest Med 44:743–755. doi:10.1016/j.ccm.2023.06.001 37890913

[12] Uwamino Y, Aoki W, Inose R, Kamoshita Y, Mikita K, Namkoong H, Nishimura T, Matsushita H, Hasegawa N. 2024. Minimum inhibitory concentrations of azithromycin in clinical isolates of Mycobacterium avium complex in Japan. Microbiol Spectr 12:e0021824. doi:10.1128/spectrum.00218-24 38687080 PMC11237530

[13] Parnham MJ, Erakovic Haber V, Giamarellos-Bourboulis EJ, Perletti G, Verleden GM, Vos R. 2014. Azithromycin: mechanisms of action and their relevance for clinical applications. Pharmacol Ther 143:225–245. doi:10.1016/j.pharmthera.2014.03.003 24631273

[14] Fröberg G, Maurer FP, Chryssanthou E, Fernström L, Benmansour H, Boarbi S, Mengshoel AT, Keller PM, Viveiros M, Machado D, et al.. 2023. Towards clinical breakpoints for non-tuberculous mycobacteria - determination of epidemiological cut off values for the Mycobacterium avium complex and Mycobacterium abscessus using broth microdilution. Clin Microbiol Infect 29:758–764. doi:10.1016/j.cmi.2023.02.007 36813087

